# COVID‐19 booster dose vaccination of healthcare workers in Qatar: A web‐based cross‐sectional survey

**DOI:** 10.1002/puh2.94

**Published:** 2023-09-04

**Authors:** Amudha Pattabi, Ananth Nazarene, Sejo Varghese, Abdulqadir Nashwan, Reena Philip, Ramya Munuswamy, Kalpana Singh

**Affiliations:** ^1^ Nursing and Midwifery Education Department Hamad Medical Corporation Doha Qatar; ^2^ Mental Health Services Hamad Medical Corporation Doha Qatar; ^3^ Department of Nursing Education and Practice Development Hazm Mebaireek General Hospital, Hamad Medical Corporation Doha Qatar; ^4^ Nursing and Midwifery Research Department Hamad Medical Corporation Doha Qatar

**Keywords:** booster dose, healthcare workers, pandemic, Qatar, vaccination, vaccine readiness

## Abstract

**Background:**

Vaccines are an important public health measure and effective strategy to protect the population from COVID‐19. Front‐line healthcare personnel should receive priority in vaccination programs. However, the reported hesitancy among healthcare workers (HCWs) toward the COVID‐19 vaccines cannot be ignored. It widely influences the level of vaccine hesitancy in the general population. Hesitancy, fear, and anxiety were documented in first and second rounds of COVID‐19 vaccination. This study assessed the acceptance of COVID‐19 booster doses among the HCWs in Qatar.

**Methods:**

A web‐based cross‐sectional online survey was conducted using the 7C Vaccine Readiness Scale to evaluate the preparedness of the HCWs to receive COVID‐19 vaccines. Descriptive and inferential statistics were used to identify factors associated with preparedness for vaccination.

**Results:**

A total of 382 participants completed the survey. Allied health professionals scored the least on the readiness score (−7.0 ± 9.9) compared to the physicians (3.1 ± 7.2) and nurses (3.0 ± 7.8). Physicians scored higher on confidence (58.8%), calculation (64.7%), and complacency (60.8%). Nurses scored higher on constraints (51.6%), collective responsibility (62.7%), and compliance (39.1%), and allied health professionals scored higher on (67.9%) conspiracy. There was a significant association between readiness score and not being infected with COVID‐19, post‐vaccine symptom experience, and hesitancy for the initial two doses.

**Conclusion:**

This study reports higher complacency and constraints with the perception of lower risks and the lack of interest in taking collective responsibility among the HCWs. Addressing vaccine hesitancy among them is critical to ensure successful vaccination campaigns and promote community safety during future pandemics.

## INTRODUCTION

With the emergence of newer variants of COVID‐19 and the reopening of socioeconomic activities to pre‐pandemic levels, vaccines are an important area of intervention worldwide [1]. Healthcare workers’ (HCWs) attitude and perception toward COVID‐19 vaccines has strong influence on their uptake among family, peers, and community, as their readiness massively influences their immediate environment either positively or negatively.

As of April 2021, there were 654,260,315 confirmed cases of COVID‐19 and 6,638,812 deaths globally, whereas Qatar reported 87,321 confirmed cases and 685 deaths [2]. Vaccination is an effective method for preventing infections and slowing the COVID‐19 virus's quick spread [3]. In the battle against COVID‐19, vaccine development and approval processes were fast‐tracked [4]. Following the United Kingdom, European Union, and Russia [5], Qatar's Ministry of Public Health (MoPH) approved Pfizer BioNTech and Moderna COVID‐19 vaccines. It distributed them among healthcare professionals, the older adults, and immune‐compromised patients through massive vaccination campaigns [6].

Vaccine hesitancy is a common issue in Arab countries, including concerns for side effects and safety [7]. A multinational study conducted before the COVID‐19 vaccine became available, involving 13,000 people, revealed that only 14% disagreed to accept a COVID‐19 vaccine if available and 18% disagreed with accepting a COVID‐19 vaccine if their employer recommended it [8]. The willingness to accept the COVID‐19 vaccine was dynamic, showing people's mind was positively [9] and negatively [10] influenced by the vaccine‐related information. A huge proportion was observed to have a “maybe” and “not sure” attitude [11], whereas one tenth of the population was not ready for vaccination [11, 12]. The concerns of the vaccinated were the post‐vaccine experience like injection site pain, tenderness, swelling, redness, tiredness, headache, myalgia, back pain, joint pain, fever, chills, sleeplessness, nausea, sore throat, diarrhea, vomiting, runny nose, and cough [13]. The fear of the side effects heard or experienced was another stumbling block toward vaccine readiness [14].

The reports on death among healthcare and frontline workers were a factor that motivated them to accept initial two doses of the COVID‐19 vaccine [15]. Factors like working with suspected or confirmed cases positioned them as a highly vulnerable section of the population compared to the general population resulting in higher rates of vaccine acceptance [15, 16, 17].

However, vaccination hesitancy among HCWs varied from 4.3% to 72% [18, 19]. COVID‐19 vaccination was mandatory in France, Germany, Italy, Greece, Finland, Croatia, the Czech Republic, Hungary, Lebanon, Poland, and New Zealand [20]. There were incidents where HCWs were banned from practice for noncompliance with the vaccine [21].

Despite successful vaccination campaign starting in December 2020, the number of cases increased in June 2021, caused by the different variants of COVID‐19 [22]. In the Arab region, the acceptance of the booster dose was relatively high among HCWs, those who had relatives infected with COVID‐19, past COVID‐19 infection, or who already received both doses of COVID‐19 vaccine [23].

After the administration of the first two rounds of vaccines and the change in the epidemiologic patterns with the emergence of new variants, there were changes and evolution in the perception and acceptance of COVID‐19 vaccines [24]. By the time the study was initiated, almost all the HCWs in Qatar were vaccinated with two doses of COVID‐19 and waiting for their booster dose. We evaluated the readiness of the HCWs in receiving booster doses of the COVID‐19 vaccine.

## METHODS

### Study design, population, and sampling

This was a web‐based cross‐sectional study based on purposive sampling among healthcare professionals of Hamad Medical Corporation (HMC), Qatar's primary healthcare provider. The study included physicians, nurses, dentists, dieticians, physiotherapists, occupational therapists, pharmacists, nurse technicians, and patient educators who had completed the first two doses of COVID‐19 vaccines. The study excluded HCWs of HMC who were not present during data collection, who took the vaccine outside Qatar, and HCWs who had already received their booster dose.

HMC employs around 28,000 HCWs [25]. The sample size was calculated for proportions at 455 with a 20% dropout rate.

### Study variables and instrument

Section I of the study instrument collected participants’ demographic details, history of the COVID‐19 infection, type of vaccine received, symptoms experienced post‐vaccination, compliance with following the COVID‐19 precautions, and the source of information for COVID‐19‐related updates. Section II contained a modified “7C Vaccination Readiness Scale” [26]. The tool assesses the confidence (trust in the security and effectiveness of vaccinations), complacency (laziness to get vaccinated due to the low perceived risk of infectious diseases), constraints (structural or psychological hurdles that make vaccination difficult or costly), calculation (the degree to which personal costs and benefits of vaccination are weighted), collective responsibility (willingness to protect others and to eliminate infectious diseases), compliance (support for societal monitoring and sanctioning of people who are not vaccinated), and conspiracy (thinking and belief in fake news related to vaccination). Every domain was assessed with a seven‐point rating scale rated between −3 and +3 [27].

### Data analysis

Data were extracted from the web‐based survey form. The 7C scores were categorized as disagree, neutral, or agree; scores were compared among three groups: nurses, physicians, and allied health professionals. Nationalities of participants were coded into five groups, the Middle East and North Africa (MENA), South Asia (India, Pakistan, and Sri Lanka), South‐East Asia (all such participants were from the Philippines), Westerners (American and British, including Australians), and African region, excluding MENA (South African, Somalian, Ugandan, Cameroon, and Nigerian).

Descriptive statistics were used to summarize demographic characteristics and data on precaution and readiness for the COVID‐19 vaccine. Precaution and readiness scores were calculated to take the sum of all the questions of respective questionnaires. *t*‐Test and ANOVA were used to compare the mean difference between the readiness score and demographic variables. Multiple linear regression was used linear regression analysis was used to determine and assess the associations between readiness score and HCWs adjusting with age, gender, education, and nationality. All statistical tests were done using STATA 17.0 with significance level at *p* < 0.05.

### Ethical considerations

The HMC Institutional Review Board (IRB) reviewed and approved the study with the study number MRC‐01‐21‐962.

## RESULTS

Three hundred eighty‐two participants had completed the survey. As shown in Table 1, the majority were aged 30–40 years (61.8%); overall, 65.7% of the participants were females, and 44.2% were allied health professionals. Table 2 describes the intentions of HCWs to accept the vaccine and their preferred sources of information on COVID‐19.

**TABLE 1:** Participant (healthcare workers) characteristics who were interviewed for COVID‐19 vaccine booster dose readiness in Qatar, 2021 (*n* = 382).

Variable	*n* (%)
**Gender**	
Male	112 (29.3)
Female	251 (65.7)
Unspecified	19 (5.0)
**Age**	
21–30 years	103 (27.0)
31–40 years	236 (61.8)
41–50 years	36 (9.4)
51 years and above	7 (1.8)
**Nationality**	
South Asia	168 (44.0)
South‐East Asia	49 (12.8)
Western	7 (1.8)
MENA	134 (35.1)
African region excluding MENA	6 (1.6)
Unspecified	18 (4.7)
**Educational qualification**	
Bachelor	284 (74.3)
Diploma	21 (5.5)
Master's	69 (18.1)
PG Diploma	4 (1.0)
PhD	4 (1.0)
**Profession**	
Nurse	161 (42.4)
Physicians	51 (13.4)
Allied health staff	168 (44.2)
**Which booster dose vaccine you are due for**	
Pfizer	296 (77.5)
Moderna	86 (22.5)

*Note*: South Asia—India, Pakistan, and Sri Lanka; South‐East Asia—Philippines; Western—America, Australia, and Britain; African region excluding MENA—South Africa, Somalia, Uganda, Cameroon, and Nigeria; MENA—Algeria, Egypt, Iraq, Jordan, Lebanon, Sudan, Syria, Tunisia, Qatar, and Palestine.

**TABLE 2:** Participants’ (healthcare workers) intentions to accept vaccine, health conditions, worries about COVID‐19, and general attitude toward vaccination in Qatar, 2021.

Variable	Nurses (*n* = 161)	Allied Health Professionals (*n* = 168)	Physician (*n* = 51)	*p* Value
**Are you a person who voluntarily takes the seasonal flu vaccine?**				
Not every year	44 (27.3)	33 (19.6)	17 (33.3)	<0.001
Agree	108 (67.1)	32 (19.0)	21 (41.2)	
Disagree	9 (5.6)	103 (61.3)	13 (25.5)	
**Have you ever been infected with COVID‐19?**				
Not infected ever	91 (56.5)	44 (26.2)	27 (52.9)	<0.001
Infected before vaccination	31 (19.3)	10 (6.0)	13 (25.5)	
Infected between two vaccines	4 (2.5)	3 (1.8)	0	
Infected after two doses of vaccination	35 (21.7)	111 (66.1)	11 (21.6)	
**Do you have any chronic illness?**				
Yes	12 (7.5)	7 (4.2)	3 (5.9)	0.44
No	149 (92.5)	161 (95.8)	48 (94.1)	
**Do you have a close relative/friend whoever infected by COVID‐19 after taking the vaccine?**				
Yes	104 (64.6)	46 (27.4)	29 (56.9)	<0.001
No	57 (35.4)	112 (72.6)	22 (43.1)	
**Do you have a close relative/friend who died due to COVID‐19 after taking the COVID‐19 vaccine?**				
Yes	21 (13.0)	16 (9.5)	1 (2.0)	0.069
No	140 (87.0)	152 (90.5)	50 (98.0)	
**Which media do you prefer to get information about COVID‐19 updates?**				
MoPH/TV news/Newspaper	128 (79.5)	155 (92.3)	40 (78.4)	0.002
Social media (Facebook, TikTok, Twitter, WhatsApp, and Telegram)	33 (20.5)	13 (7.7)	11 (21.6)	
**Did you have any hesitation to receive the first dose of the COVID‐19 vaccine?**				
Yes	34 (21.1)	109 (64.9)	14 (27.5)	<0.001
No	127 (78.9)	59 (35.1)	37 (72.5)	
**Did you have any hesitation to receive the second dose of the COVID‐19 vaccine?**				
Yes	20 (12.4)	106 (63.1)	8 (15.7)	<0.001
No	141 (87.6)	62 (36.9)	43 (84.3)	
**Did you experience any symptoms after your COVID‐19 vaccine?**				
Yes	96 (59.6)	39 (23.2)	24 (47.1)	<0.001
No	46 (28.6)	107 (63.7)	14 (27.5)	
Not sure	19 (11.8)	22 (13.1)	13 (25.5)	

Table 3 presents readiness to accept COVID‐19 booster doses based on the 7C framework. Nurses (54%) and physicians (60.8%) scored higher on complacency compared to allied health professionals. Nurses scored higher (51.6%) on constraints. Physicians (64.7%) scored higher on calculation. Nurses scored higher on collective responsibility (62.7%) and compliance (39.1%). Allied health professionals scored higher on (67.9%) conspiracy.

**TABLE 3:** COVID‐19 booster dose vaccination readiness among healthcare professionals, Qatar 2021.

Variable	Nurse (*n* = 161) (%)	Allied Health professionals (*n* = 168) (%)	Physicians (*n* = 51) (%)	*p* Value
**Confidence**				
Agree	79 (49.1)	35 (20.8)	30 (58.8)	<0.001
Neutral	36 (22.4)	24 (14.3)	12 (23.5)	
Disagree	46 (28.6)	109 (64.9)	9 (17.6)	
**Complacency**				
Agree	87 (54.1)	36 (21.4)	31 (60.8)	<0.001
Neutral	34 (21.1)	27 (16.1)	9 (17.6)	
Disagree	40 (24.8)	105 (62.5)	11 (21.6)	
**Constraints**				
Agree	83 (51.6)	38 (22.6)	24 (47.1)	<0.001
Neutral	40 (24.8)	25 (14.9)	15 (29.4)	
Disagree	38 (23.6)	105 (62.5)	12 (23.5)	
**Calculation** **^a^**				
Agree	95 (59.0)	42 (25.0)	33 (64.7)	<0.001
Neutral	35 (21.7)	27 (16.1)	10 (19.6)	
Disagree	31 (19.3)	99 (58.9)	8 (15.7)	
**Collective responsibility**				
Agree	101 (62.7)	48 (28.6)	30 (58.8)	<0.001
Neutral	31 (19.3)	19 (11.3)	13 (25.5)	
Disagree	29 (18.0)	101 (60.1)	8 (15.7)	
**Compliance**				
Agree	63 (39.1)	27 (16.1)	18 (35.3)	<0.001
Neutral	56 (34.8)	28 (16.7)	18 (35.3)	
Disagree	42 (26.1)	113 (67.2)	15 (29.4)	
**Conspiracy** **^a^**				
Agree	39 (24.2)	114 (67.9)	10 (19.6)	<0.001
Neutral	51 (31.7)	23 (13.7)	16 (31.4)	
Disagree	71 (44.1)	31 (18.5)	25 (49.0)	

^a^ Question is negatively worded.

Abbreviation: MENA, Middle East and North Africa.

Table 4 shows the association between readiness for COVID‐19 booster dose uptake with demographic variables. Higher readiness scores for COVID‐19 booster dose were noted among females (0.8 ± 9.0), those older than 51 years (4.1 ± 7.8), and those originating from the African region excluding MENA (2.7 ± 7.3). Allied health professionals had the least readiness score (−7.0 ± 9.9) as compared to nurses (3.0 ± 7.8) and physicians (3.1 ± 7.2).

**TABLE 4:** Association between demographic variables of healthcare professionals and COVID‐19 booster dose vaccination readiness, Qatar 2021 (*n* = 382).

Variable	*n* (%)	Mean ± SD	*p* Value
**Gender**			
Male	112 (29.3)	−4.2 ± 10.6	<0.001
Female	251 (65.7)	0.8 ± 9.0	
Unspecified	19 (5.0)^a^		
**Age**			
21–30 years	103 (27.0)	3.4 ± 6.8	<0.001
31–40 years	236 (61.8)	−3.7 ± 10.8	
41–50 years	36 (9.4)	−1 ± 7.4	
51 years and above	7 (1.8)	4.1 ± 7.8	
**Nationality**			
South Asia	168 (44.0)	0.5 ± 10.2	<0.001
South‐East Asia	49 (12.8)	−2.7 ± 12.1	
Western	7 (1.8)	0.7 ± 5.7	
MENA	134 (35.1)	−1.8 ± 8.5	
African region excluding MENA	6 (1.6)	2.7 ± 7.3	
Unspecified	18 (4.7)^a^		
**Educational qualification**			
Bachelor	284 (74.3)	−0.8 ± 10.1	0.005
Diploma	21 (5.5)	1.6 ± 8.9	
Master's	69 (18.1)	‐5.3 ± 9.3	
PG Diploma	4 (1.0)	1.5 ± 8.7	
PhD	4 (1.0)	3.8 ± 9.0	
**Profession**			
Nurse	161 (42.1)	3.0 ± 7.8	<0.001
Physicians	51 (13.4)	3.1 ± 7.2	
Allied health staff	168 (44.0)	−7.0 ± 9.9	
Unspecified	2 (0.5)*		
**Which booster dose vaccine you are due for?**			
Pfizer	296 (77.5)	−2.3 ± 10.3	0.002
Moderna	86 (22.5)	1.6 ± 8.6	
**Do you have any chronic illness?**			
Yes	23 (6.0)	2.0 ± 9.8	0.095
No	359 (94.0)	−1.6 ± 10.0	
**Did you have any hesitation to receive the first dose of the COVID‐19 vaccine?**			
Yes	159 (41.6)	−8.7 ± 8.6	<0.001
No	223 (58.4)	3.8 ± 7.4	
**Did you have any hesitation to receive the second dose of the COVID‐19 vaccine?**			
Yes	136 (35.6)	−10.8 ± 7.2	<0.001
No	246 (64.4)	3.8 ± 7.2	
**Have you ever been infected with COVID‐19?**			
Not infected ever	164 (42.9)	3.1 ± 7.7	<0.001
Infected before vaccination	54 (14.1)	3.0 ± 7.5	
Infected between two vaccines	7 (1.8)	0.1 ± 10.9	
Infected after two doses of vaccination	157 (41.1)	−7.6 ± 9.7	
**Did you experience any symptoms after your COVID‐19 vaccine?**			
Yes	160 (41.9)	1.9 ± 8.4	<0.001
No	167 (43.7)	−5.6 ± 11	
Not sure	55 (14.4)	1.8 ± 5.4	
**Are you a person who voluntarily takes the seasonal flu vaccine?**			
Not every year	94 (24.6)	2.2 ± 7.2	<0.001
Agree	163 (42.7)	3.3 ± 7.8	
Disagree	125 (32.7)	−10.2 ± 8.6	
**Which media do you prefer to get information about COVID‐19 updates?**			
MoPH/TV news/News paper	324 (84.8)	−2.2 ± 10.2	<0.001
Social media (Facebook, TikTok, Twitter, WhatsApp, and Telegram)	58 (15.2)	2.9 ± 8.1	
**Do you have a close relative/friend whoever infected by COVID‐19 after taking the vaccine?**			
Yes	179 (46.9)	2.4 ± 8.2	<0.001
No	203 (53.1)	−4.7 ± 10.4	
**Do you have a close relative/friend who died due to COVID‐19 after taking the COVID‐19 vaccine?**			
Yes	38 (10.0)	−0.2 ± 8.8	0.450
No	344 (90.0)	−1.5 ± 10.2	

^a^ Data with missing values (unspecified), mean was not calculated.

The study showed a huge readiness gap among the participants who were never infected with COVID‐19 (3.1 ± 7.7), those who had suffered (1.9 ± 8.4) from post‐vaccine symptoms, and not suffered from symptoms (−5.6 ± 11.0). The participants who had hesitancy for the first two doses of the COVID‐19 vaccine had low scores (−8.7 ± 8.6 and −10.8 ± 7.2), respectively. HCWs who referred social media (2.9 ± 8.1) for COVID‐19‐related information and HCWs had witnessed their vaccinated relatives/friends who were infected with COVID‐19 (2.4 ± 8.2) had higher readiness scores.

**FIGURE 1:**
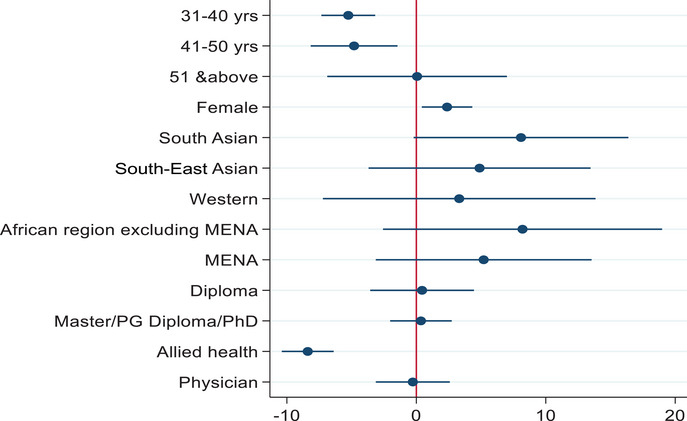
Scores for readiness in the uptake of COVID‐19 booster doses among healthcare professionals in Qatar, 2021 adjusted for age, gender, education, profession, and nationality.

A multiple linear regression showed that HCWs aged 31–40 and 41–50 years, and allied health professionals had significantly lower readiness scores (Figure 1). The readiness for the COVID‐19 vaccination was higher in females (coefficient 2.37; 95%CI: 0.43–4.32). No significant association was found in terms of nationality and education.

## DISCUSSION

Addressing vaccine hesitancy among HCWs is critical to ensure successful vaccination campaigns and promote community safety during future pandemics. Prioritizing efforts to build trust and improve communication between HCWs and health authorities is necessary to increase vaccine readiness and uptake. Failure to address vaccine hesitancy among HCWs may hinder future efforts to protect public health, emphasizing the importance of taking action to promote vaccine acceptance and address concerns.

This is probably the first comprehensive study in the Middle Eastern region to investigate HCW readiness for COVID‐19 vaccine booster dose. The study was able to capture and survey many healthcare professionals working for Qatar's main health provider. The use of the 7C instrument makes its findings comparable to other studies [28].

The trust and readiness for the uptake of COVID‐19 booster doses varied among the HCWs in Qatar. Most physicians agreed that they took the vaccine because they felt it was too risky (complacency) to be infected. One third of nurses and physicians agreed on mandating vaccination, including sanctions for noncompliance similar to that reported in Poland [29]. Allied health workers reported COVID‐19 booster doses are not their priority (“constraints”). The same attitude trend was reported among the HCWs in Italy [30]. Although more than half of the nurses agreed on taking the vaccine with the willingness to protect others and thus eliminate COVID‐19 (“collective responsibility”), allied health workers disagreed on the same. The same was reported from China, South Korea, India, Brazil, and Ecuador [31]. The overall disagreement on “collective responsibility” in non‐mandated vaccination campaigns was 31.2% compared to 52.7% in Poland and 52.5% in Russia [31].

The study showed a huge readiness gap between HCWs who were not infected ever with COVID‐19 and those who had shown hesitancy for the first two doses of COVID‐19 vaccine [32] reflecting the persistence of risk perception. HCWs who had suffered from post‐vaccine symptoms were noted to have high readiness scores that may be explained by their perceived occupational exposure and risk for infection [33]. HCWs who witnessed the suffering of their relatives/friends who were infected with COVID‐19 even after vaccination also showed more readiness as reported in an Italian study [34].

Most participants, especially allied health workers, relied upon the MoPH and World Health Organizations’ (WHO) official pages for COVID‐19 updates. These findings are well similar to an Ethiopian study [35], which reported statistical significance on their readiness [36] toward booster dose. However, some studies reported that social media were the priority choice for information about COVID‐19 among HCWs [37].

Allied health professionals had the least readiness score (−7.0 ± 9.9), whereas the physicians’ vaccination readiness score was high (3.1 ± 7.2), the same was reported by a French study [38]. Although this study reported the significant association between readiness score and age, sex, and profession, another study reported non‐significance [39].

### Limitations of the study

There was an attempt to reduce the likelihood of desirability bias through the anonymous online questionnaire (e.g., respondents’ names and precise work locations were not asked for) and voluntary participation. However, the relatively small size of the participants compared to the total HCWs of HMC may raise concerns over the generalization. The questionnaire was designed to collect mandatory responses before moving to the next page until submission. However, to mitigate the survey fatigue, the questionnaire was prepared with simple drop‐down options to choose from.

## CONCLUSIONS

This study reports significant differences in the levels of readiness among different categories HCWs in their readiness toward the uptake of booster doses of the COVID‐19 vaccine. Some of the challenges in the uptake of booster doses are high levels of complacency and constraints with the perception of lower risks and the lack of interest in taking collective responsibility. The levels of readiness in HCWs have major influence on the attitudes toward vaccines in the general population.

## AUTHOR CONTRIBUTIONS

All authors were involved in the design of the work or the acquisition, analysis, or interpretation of data. In addition, all authors contributed to writing and/or revising the article.

## CONFLICT OF INTEREST STATEMENT

The authors declare that they have no conflict of interest.

## ETHICS STATEMENT

The HMC Institutional Review Board (IRB) reviewed and approved the study with the study number MRC‐01‐21‐962 on 09/12/2021.

## PARTICIPANT'S CONSENT STATEMENT

As per inclusion and exclusion criteria, a survey link was sent to all eligible staff in HMC with the subject information sheet and a questionnaire with Sections I and II. Those who completed the questionnaire were considered as voluntarily giving consent to participate in the study.

## PERMISSION TO REPRODUCE MATERIAL FROM OTHER SOURCES

Official permission was sought through Email, and the author was cited for the same in the manuscript.

## Data Availability

The data that support the findings of this study are available from the corresponding author upon reasonable request.
